# Whole genome sequence of Paracoccus sp. NFXS7, a carotenoid-producing bacterium isolated from a marine saltern

**DOI:** 10.1099/acmi.0.000862.v2

**Published:** 2024-07-23

**Authors:** Constança D.F. Bertrand, Rodrigo Martins, Francisco Quintas-Nunes, Pedro Reynolds-Brandão, Maria T. Barreto Crespo, Francisco X. Nascimento

**Affiliations:** 1 iBET, Instituto de Biologia Experimental e Tecnológica, Apartado 12, 2781-901 Oeiras, Portugal; 2 ITQB-NOVA, Instituto de Tecnologia Química e Biológica António Xavier, Universidade Nova de Lisboa, Av. da República, 2780-157 Oeiras, Portugal

**Keywords:** carotenoid, genome, marine, *Paracoccus*

## Abstract

The study presents the whole genome sequence of the carotenoid-producing *Paracoccus* sp. NFXS7, isolated from a marine saltern in Setúbal, Portugal. The carotenoid-producing strain NFXS7 contains homologs of the *crt* genes involved in astaxanthin biosynthesis, making it a promising candidate for biotechnological applications.

## Data Summary

This Whole Genome Shotgun project has been deposited in the National Center for Biotechnology Information (NCBI) under the accession number JAKEKV000000000.1. The whole genome sequence reads used can be accessed in the NCBI’s Sequence Read Archive under the accession number PRJNA795090 (https://www.ncbi.nlm.nih.gov/sra/PRJNA795090).

The authors confirm all supporting data, code and protocols have been provided within the article or through supplementary data files.

## Introduction

Marine environments are rich in biodiversity and serve as a valuable resource for the discovery of novel microbial strains of industrial relevance. Among these, *Paracoccus* has gained significant attention due to its ability to produce carotenoids [1,2], a class of pigments with potential applications in various industries, including food, pharmaceuticals and cosmetics [3]. Several species of *Paracoccus* (*Paracoccus carotinifaciens,* the most studied) produce high yields of carotenoids such as astaxanthin, which makes them suitable for industrial applications [4,6]. Still, despite the known biotechnological potential of *Paracoccus*, not much is understood about the genomic properties of these strains, the genetic mechanisms regulating carotenoid biosynthesis, and the synthesis of other relevant secondary metabolites.

In this study, we report the genome sequence of a novel carotenoid-producing *Paracoccus* strain (NFXS7) isolated from marine saltern water in Setúbal, Portugal (38°29′35.0″ N 8°46′26.4″ W), bringing new insights into the genomic properties of carotenoid-producing *Paracoccus*.

## Methods

The strain was isolated using marine agar (Condalab, Spain) and was selected due to its deep orange coloration. Strain NFXS7 was routinely cultivated in marine agar and marine broth at 26 °C (at 180 r.p.m. when cultivated in liquid broth). Total DNA was isolated from an overnight grown culture using the PureLink Genomic DNA Kit (Invitrogen, USA) according to the manufacturer’s instructions. The bacterial DNA was sent to MicrobesNG (https://microbesng.com/) (UK) and processed under the company-defined pipeline (https://microbesng.com/documents/methods/). The genomic DNA libraries were prepared using the Nextera XT Library Prep Kit (Illumina, San Diego, USA) following the manufacturer’s protocol with the following modifications: input DNA was increased twofold, and PCR elongation time was increased to 45 s. The DNA quantification and library preparation were conducted using a Hamilton Microlab STAR automated liquid handling system (Hamilton Bonaduz AG, Switzerland). The libraries were sequenced using lllumina NovaSeq 6000 (Illumina, San Diego, USA) using a 250-bp paired-end protocol. The obtained reads were trimmed using Trimmomatic version 0.30 [7] with a sliding window quality cutoff of Q15. A total of 1 682 720 reads with a median insert size of 642 bp were obtained. The genome sequence of strain NFXS7 was assembled using the SPAdes v 3.15.5 [8] and annotated using the National Center for Biotechnology Information Prokaryotic Annotation Pipeline [9]. The functional annotation of the genome was performed using BlastKOALA [10]. Secondary metabolite clusters were predicted using the antiSMASH version 7.1.0 [11].

## Genome Description and Future Outlook

The whole genome sequence assembly of *Paracoccus* sp. NFXS7 comprises 35 contigs (N50 = 296 487) totaling a genome size of 3 845 849 bp (3.845 Mbp) and a GC of 67%. The total genome coverage was 103X. A total of 3767 genes were identified, of which 3626 are complete coding sequences and 50 are encoding RNA-related genes. The BlastKOALA functional annotation resulted in 2097 entries (57.8%), mostly involved in genetic information processing (408), environmental information processing (278), carbohydrate metabolism (232) and signalling and cellular processes (201), followed by the metabolism of amino acids (127), cofactors and vitamins (113) and nucleotides (84). The antiSMASH annotation revealed the presence of gene clusters involved in the biosynthesis of ectoine, a siderophore, a carotenoid, an unknown Non-ribosomal peptide synthase and *N*-acyl-l-homoserine lactones. Additionally, analysis of the NFXS7 carotenoid biosynthetic cluster (*di, crtWZYIBEX*) showed that it presented a high similarity (97.39%) to the functional carotenoid biosynthetic cluster of *Paracoccus* sp. N81106 (GenBank accession: AB206672.1) is involved in the biosynthesis of astaxanthin and its intermediates [12].

The whole genome sequence assembly of *Paracoccus* sp. NFXS7 provides a valuable resource for future research aiming to understand *Paracoccus* physiology, secondary metabolite biosynthesis and stress resistance mechanisms, which are the key factors for the development of novel potential biotechnological applications.

## Abbreviation

NCBI: National Center for Biotechnology Information
